# Influenza A/H3N2 epidemiology in England during the 2025 to 2026 season: a mathematical modelling study

**DOI:** 10.1038/s44528-026-00016-3

**Published:** 2026-08-05

**Authors:** James A. Hay, Punya Alahakoon, Alexander Greenshields-Watson, Michelle Kendall, Mahan Ghafari, Chris Wymant, Robert Hinch, Luca Ferretti, Jasmina Panovska-Griffiths, Christophe Fraser

**Affiliations:** 1 Pandemic Sciences Institute, Nuffield Department of Medicine, University of Oxford, Oxford, UK; 2 Department of Biology, University of Oxford, Oxford, UK; 3 The Queen’s College, University of Oxford, Oxford, UK; 4 UK Health Security Agency, London, UK

**Keywords:** Computational biology and bioinformatics, Diseases, Immunology, Microbiology

## Abstract

**Background:**

England experienced an unusually early and rapid increase in influenza A/H3N2 subclade K infections in 2025/26. Antigenic change and a fast selective sweep raised concerns over a potentially severe season. Building on analysis conducted as the subclade emerged, we aim to compare epidemic dynamics of the 2025/26 season to previous years and to model plausible epidemiological scenarios.

**Methods:**

We compared peak epidemic growth rates and reproduction numbers across influenza seasons from 2011/12 to 2025/26 using routine surveillance data in England. Weekly epidemic growth rates were estimated using a Gaussian random walk model, and time-varying reproduction numbers using *EpiEstim*. We also developed an age-stratified transmission model and interactive web tool to explore scenarios varying immune escape, transmissibility, and seed date, using 2022/23 as a baseline season.

**Results:**

Peak A/H3N2 growth rates and time-varying reproduction numbers for the 2025/26 season are of similar magnitude but earlier than previous severe seasons. Scenario analyses suggest early trends are compatible with moderate levels of immune escape, a 10% higher *R*_*0*_, or an earlier seed date, though it is not possible to distinguish the relative importance of these mechanisms from these data alone.

**Conclusions:**

The 2025/26 influenza season is characterised by early but not unusually rapid growth. Earlier growth does not systematically lead to especially large epidemics due to earlier susceptible depletion combined with a dampening effect from school holidays. Laboratory evidence for antibody escape does not directly translate to large reductions in population immunity, supporting the need for complementary real-time epidemiological analyses and modelling.

## Introduction

The 2025/2026 influenza season in the northern hemisphere was dominated by an antigenically drifted subclade of A/H3N2 viruses. This K subclade descended from the J.2 clade on which the vaccine strain selection was based and swept to dominance from a frequency of <1% on 2025-07-02 to 94% in Europe as of 2025-12-13^1^. This suggested a substantial fitness advantage over other J.2 viruses^2^. The combination of rapid growth, multiple antigenic substitutions in the haemagglutinin (HA) protein, antigenic mismatch with the vaccine strain, typically higher morbidity and mortality amongst the elderly during A/H3N2 seasons^3^, and an unusually early start raised concerns over a potentially severe season^4–6^. Furthermore, the 2025 flu season in Australia was one of the worst on record, with an unusually long tail driven by subclade K^7^, and Japan suffered from an early epidemic leading to school closures and increased hospitalisations^8^.

The interaction of season timing, antigenic drift, other subtype dynamics, vaccine efficacy and coverage, non-HA-mediated immunity, immune waning from previous seasons, climate factors, and contact pattern changes around school holidays is complex and leads to highly varied cumulative and peak seasonal burden^9^. The potential outlook of the 2025/2026 influenza season was therefore uncertain based on early epidemiological data, though historical seasons with early onset have often been severe^3^.

Real-time epidemiological assessment in England is based on weekly influenza surveillance data from the Second-Generation Surveillance System (SGSS)^11^, which compiles respiratory virus testing data from multiple sources: the Royal College of General Practitioners (RCGP) Research & Surveillance Centre (RSC) (primary care), the Respiratory DataMart system (hospital testing) and other hospital testing. The main surveillance indicator in the UK Health Security Agency (UKHSA) weekly surveillance reports is the percentage of all tests in the SGSS that are positive for influenza among all patients presenting with influenza-like illness (ILI). This metric increased much earlier than in other recent seasons and started declining as of the report published on 2025-12-18 using data up to 2025-12-14, and has continued to decline based on data reported up to 2026-03-30^10,11^. An advantage of the percentage positive metric is that it is thought to be less biased by changes in overall testing volume than count data; however, it is also affected by the dynamics of other ILI-causing pathogens. An ILI+ indicator, which multiplies ILI cases by the percentage of tests positive for influenza, is therefore often recommended^12^.

To understand the transmission rate and possible dynamics of the subclade K viruses in England, we analysed publicly available epidemiological data from England and developed an age-stratified Susceptible-Infected-Recovered model to separately address two questions: (1) are epidemic growth rates and the time-varying reproduction number, *R*_*t*_, from the 2025/26 influenza season indicative of a more transmissible A/H3N2 strain than in previous seasons?; and (2) under plausible scenarios of a strain with increased transmissibility, earlier seeding, and/or substantial immune evasion, what is the potential impact on cumulative and peak influenza infection incidence relative to previous seasons? We find that the peak epidemic growth rate of the 2025/2026 influenza season is high but not exceptional, comparable to previous seasons in the past 15 years. Scenario modelling analysis suggests that, as of 2025-12-18, the epidemic trajectory could be consistent with at most a modest reduction in population immunity compared to previous years unless compensated by large changes to other epidemiological parameters, or an earlier epidemic seed time. These analyses were prompted by the uncertainty about the early appearance of the new subclade in the UK and accompanying data on immune escape. The paper builds on analyses presented to the UK Department of Health in November 2025.

## Methods

### Epidemiological analyses

#### Data summary

We analysed weekly counts of reported influenza cases by subtype for England from the Respiratory DataMart system from 2009-04-27 to 2025-12-08 (Fig. S1). We also analysed weekly counts of reported influenza specimens stratified by subtype for England from the WHO FluNet platform between 2011-01-09 and 2025-12-14^13^. All data are aggregated by week, and we used the first day of the epidemiological week as the reported date. We removed the most recent week of data to avoid issues arising from reporting delays. As many influenza A cases are not subtyped, we distributed unsubtyped counts into influenza A/H3N2 and A/H1N1pdm09 counts proportional to the ratios of subtyped samples.

To understand age-specific dynamics, we also used data on influenza cases by age group and by subtype from the Royal College of General Practitioners Research & Surveillance Centre (RCGP RSC) and ILI from the RCGP RSC weekly reports (Figs. S1–3). Following recommendations from^12^, we implemented an ILI+ indicator for each age group as:

1 $${{ILI}}_{+}={ILI}* {p}_{+{ve}}* N$$

Where ${p}_{+{ve}}$ is the proportion of all tests which are positive for influenza either overall or by subtype and N is the number of individuals in that age group. Note that imperfect case ascertainment is implicit in the ILI data.

### Second-Generation Surveillance System (SGSS)

Weekly influenza positivity from PCR tests by age was obtained from the UKHSA dashboard for the SGSS, available at ref. ^14^. Values are shown as a percentage of people with at least one positive PCR result for influenza (in a 7-day period). Further details are available at ref. ^15^. This dataset provided us with the proportion of tests that are positive for influenza, but not the absolute number of tests performed, the proportion positive stratified by influenza subtype, nor stratification by age.

### Royal College of General Practitioners (RCGP) Research & Surveillance Centre (RSC)

The RCGP RSC is a nationally representative primary care-based surveillance system. Weekly communicable and respiratory disease reports are publicly available at ref. ^16^. We compiled weekly national incidence of influenza-like illness (ILI) rates (per 100,000 population) for England. PDF reports were downloaded at 6-month intervals from January 2021 to December 2025, and Table E from each report was converted to CSV. These tables included age-stratified ILI rates across four age categories differing by reporting period:

1. 1–4 years, 5–14 years, 15–64 years and 65+ years, and all ages from week 26, 2023 to week 44, 2025.
2. <15 years, 15–64 years, 65+ years, and all ages for weeks 40–53, 2020, week 1, 2021 to week 5, 2021, and weeks 1–26, 2023.

This dataset provides ILI rates by age, which we convert to estimates for absolute ILI case numbers by multiplying by the population size per age group^17^. To align different age bands from the two reporting periods, we redistributed the older ILI rates and percentage positive for influenza into the more recent age bands. First, we expanded age bands into per-year groups, assuming that the value reported within an age group applied to all ages within that group. We then recombined these per-year variables into common age bands (1–4, 5–14, 15–64 and 65+ years), either by summing counts and denominators within the new age band for incidence data, or by calculating the population-weighted mean using the per-year age distribution of England for positivity data^17^.

We also digitised data from the RCGP Virology Dashboard on the total number of samples tested and the number of samples positive for influenza by age group and by subtype for the 2022/2023 to 2025/2026 influenza seasons^18^.

### Respiratory DataMart sentinel system for hospital testing

The Respiratory DataMart is a sentinel laboratory surveillance system which monitors all major respiratory viruses in England, compiling data from 17 contributing laboratories^19^. Participating laboratories test swabs for respiratory viruses, including influenza A viruses, using real-time polymerase chain reaction (RT-PCR), though not all laboratories test for or report all viruses. The absolute number of samples in this system is much lower than SGSS, but it gives absolute numbers of tests rather than just percentage positive.

We created a combined dataset spanning the period 27 April 2009 to 8th December 2025^14^. This included weekly counts of laboratory-confirmed influenza cases by subtype: influenza A (not subtyped), influenza A(H1N1)pdm09, influenza A(H3N2), and influenza B. Additionally, the dataset provides the overall percentage of specimens testing positive for influenza. However, these data were not stratified by age group.

### World Health Organisation (WHO) FluNet

Weekly counts of reported influenza specimens were obtained from the WHO FluNet platform^13^. This is the global influenza virological surveillance system collecting weekly sentinel and non-sentinel (e.g., outbreak investigations, point-of-care testing) surveillance data from national influenza centres. Data were extracted for the period 9th January 2011 to 14th December 2025, restricted to England. The extracted dataset included the weekly number of samples classified as A(H3), influenza A (not subtyped), A(H1N1pdm09) influenza B, and overall influenza cases. We allocated the unsubtyped influenza A cases into A/H3N2 and A/H1N1 cases proportional to the ratio of subtyped samples.

### Weekly growth rate calculations

Empirical weekly growth rates were calculated for each influenza season and aligned by calendar week as:

2 $$y=log \left(\frac{i\left(t\right)}{i\left(t-1\right)}\right)$$

where i(t) is the reported incidence over week *t*. We calculated smoothed weekly exponential growth rates of influenza cases in England from the Respiratory DataMart using a Gaussian random walk model fitted to i(t) as described by Eales et al.^20^. Note that this model uses a prior distribution which penalises changes in the first derivative (approximately the growth rate). We also calculated overall weekly growth rates using data from the WHO FluNet for comparison using the same method. Finally, we fitted a Generalised Additive Model (GAM) to i(t) with a thin-plate spline using the *mgcv* R package to describe smoothed weekly growth rates of the age-stratified ILI+ data from the RCGP RSC data^21^.

### Time-varying reproduction number estimation

We estimated the time-varying reproduction number (*R*_*t*_) using the *EpiEstim* package in R^22^. *R*_*t*_ is defined as the expected number of new infections at time *t*, divided by past incidence weighted by relative infectiousness given by the generation interval distribution. *EpiEstim* uses an expectation-maximisation algorithm to estimate parameters of the following renewal equation given incidence data^22,23^:

3 $${I}_{t} \sim {Pois}\left({R}_{t}\mathop{\sum }\limits_{s=1}^{t}{I}_{t-s}{g}_{s}\right)$$

Where ${I}_{t}$ denotes the case incidence at time *t*; ${I}_{t-s}$ is the past incidence; $\mathrm{Pois}$ refers to the Poisson distribution; and ${g}_{s}$ is the probability mass of the serial interval distribution at time-since-infection *s*.

We used the overall weekly influenza incidence data for the period 2011/2012 season to the 2025/2026 season from the UKHSA Respiratory DataMart for this analysis. To approximate daily incidence, weekly counts were distributed evenly across days per week and then smoothed with a 14-day rolling mean. The serial interval distribution was assumed to follow a distribution with a mean of 3.6 days and a standard deviation of 1.6 days, based on ref. ^24^. We used a window size of 14 days and the default Gamma-distributed prior on *R*_*t*_ with a mean of 5 and standard deviation of 5^22^.

### Compartmental model and scenario analyses

#### Model overview

To explore potential epidemic scenarios distinguishing the current influenza season from the previous A/H3N2 season in 2022/2023, we simulated seasonal influenza transmission dynamics for England using an age- and immunity-structured deterministic Susceptible-Infected-Recovered model. We explored scenarios where the K subclade viruses exhibit enhanced transmissibility, greater immune escape, or an earlier epidemic seed time. We compared the impact of these scenarios on the epidemic timing around the half-term and Christmas school holidays, the final epidemic size, the final size in 65+ year olds, and peak incidence as a proxy for maximum healthcare burden, assuming that peak infection incidence is proportional to peak hospitalisation rate. We emphasise that we did attempt to formally compare model-predicted growth rates to observed growth rates based on estimates from the “Epidemiological analysis” section.

#### Model structure

We divided the population into four age groups (0–4, 5–18, 19–64 and 65+ years) and two immunity classes (fully susceptible and partially immune), representing eight classes in total.

The force of infection in group *i* was defined as:

4 $${\lambda }_{i}\left(t\right)=\beta \mathop{\sum }\limits_{j=1}^{m}{C}_{i,j}\left(t\right){I}_{j}\left(t\right)$$

Where *β* is the overall transmission rate (not age-stratified), *C*_*i,j*_ is the contact rate between group *i* and group *j*, and *I*_*j*_ is the number of infected individuals in group *j* at time *t*. The basic reproduction number, *R*_0_, was given by the largest eigenvalue of the next-generation matrix with contact matrix *C*, multiplied by *β* and the infectious period, *T*_*g*_. The model assumes random mixing between individuals (mass action).

Transition rates between the three compartments were defined by the following set of ordinary differential equations:

5 $$\frac{d{S}_{a,k}}{{dt}}=-{\eta }_{k}{S}_{a,k}\left(t\right){\lambda }_{a,k}\left(t\right)$$

6 $$\frac{d{I}_{a,k}}{{dt}}={\eta }_{k}{S}_{a,k}\left(t\right){\lambda }_{a,k}\left(t\right)-\frac{{I}_{a,k}\left(t\right)}{{T}_{g}}$$

7 $$\frac{d{R}_{a,k}}{{dt}}=\frac{{I}_{a,k}\left(t\right)}{{T}_{g}}$$

Where ${\eta }_{k}$ denotes the relative susceptibility of immune class *k* and *T*_*g*_ is the infectious period. ${\eta }_{k}$ is a scaling factor which modulates the force of infection, ${\lambda }_{a,k}\left(t\right)$, for age group *a* and immune class *k*, where values less than 1 represent partial protection and values greater than 1 represent enhanced susceptibility. We modelled two immunity classes, $k\in \{\mathrm{1,2}\}$ where k=1 represents the susceptible class and k=2 represents the immune class. We set ${\eta }_{1}$ to 1 for the susceptible population and ${\eta }_{2}$ to 0 for the immune population, representing all-or-nothing immunity (i.e., all immune individuals at baseline are unable to become infected). Note that ${\eta }_{k}$ is therefore effectively not used in the model but is included for completeness and to allow extension to scenarios assuming leaky immunity.

Overall immune escape was modelled as a single parameter, *δ*, which scales the initial population immune proportion in all age groups (*δ* = 0 corresponds to complete immune escape, whereas *δ* = 1 corresponds to no loss of population immunity relative to baseline). This parameter is used to scale the initial model conditions as:

8 $${N}_{a,1}\left(0\right)=\left(1-\delta \right){N}_{a},{N}_{a,2}\left(0\right)={\delta N}_{a}$$

${N}_{a,k}\left(0\right)$ is then split across ${S}_{a,k}\left(0\right)$, ${I}_{a,k}\left(0\right)$, and ${R}_{a,k}\left(0\right)$ based on the proportions shown in Table S1. We solved the model in daily timesteps using the *deSolve* R package^25^.

### Baseline model calibration

We used our intuition and manual calibration to generate a baseline scenario with epidemiological dynamics similar to what was seen in the 2022/2023 season. As a starting point, initial model parameters were based on standard seasonal influenza parameter values (*R*_0_ of around 2, infectious period of 4–5 days, and final size of around 15%^26,27^). We then manually adjusted the model parameters to broadly align the model-predicted influenza case incidence curves with the age-stratified ILI+ data from the 2022/2023 season based on four criteria: (1) the date of peak reported cases; (2) the magnitude of peak reported cases; (3) the cumulative number of reported cases; and (4) ensuring that the overall final size was between 15% and 20%. We also calculated the root mean squared error (RMSE) and RMSE normalised by the peak in reported cases between the model-predicted incidence curves and observed data. We note that this is a complex model with a large number of highly correlated parameters, making formal model fitting difficult. Many of the parameters are hard to identify and interpret, such as the overall fraction of symptomatic cases reported, the symptomatic fraction by age combined with age-specific reporting rates, and the level of immune escape of the seed virus. Thus, we focused on comparing outputs such as the timing and relative magnitude of peak reported cases and the overall proportion infected to the baseline 2022/2023 scenario, rather than the growth rate or precise parameter inference, by varying a limited number of key parameters. Parameter values used for the baseline scenario are shown in Table S1.

We set the population size of the model to 60,000,000 to approximate the population size of England. We distributed the population into age groups based on the age distributions in the *socialmixr* package using the POLYMOD data^28^. Each age group was stratified into the susceptible or fully immune class, assuming different levels of immune escape for each age group. The epidemic was seeded by arbitrarily setting *I*_19-64_(0) = 1000; the seed time was varied during model calibration.

### Contact matrices over time

Symmetric, age-stratified contact matrices were generated using all contact data from the POLYMOD UK study using the *socialmixr* R package^28^. We generated four contact matrices for different time periods: (1) regular school term-time; (2) half-term with no school contacts and reduced school contacts; (3) pre-Christmas shopping period (2022-12-01 to 2022-12-21) with an increase in all non-school contacts; and (4) the Christmas school holiday period (2022-12-21 to 2023-01-05) with no school contacts, a reduction in all contacts, and an increase in at-home contacts. These matrices were constructed by resampling the original POLYMOD contact diary entries with replacement and applying multipliers for home, work and other contact types. We smoothed the transition between contact matrices over 3 days before and after the holiday period using a cosine function. Holiday dates were based on the Oxfordshire school holiday period. For all non-school contacts, the relative changes in contacts over half-term holidays and the Christmas period (shopping and holiday) were extrapolated from 2021–2023 data from Kendall et al.^29^. Absolute changes in overall non-school contact rates were extrapolated from the same source.

### Scenario analyses

Scenario analyses were chosen to illustrate potential hypotheses for the early and rapid growth of A/H3N2 cases in England for the 2025/2026 season. We also varied the immune escape scaling parameter *δ* (testing all values from 0 to 1 in increments of 0.01), the basic reproduction number *R*_0_ (testing all values from 1.01 to 3 in increments of 0.01), the proportion of the 0–4 and 5–18 year old population initially immune (testing all values from 0 to 1 in increments of 0.01), and the seed date univariably (testing seed dates from 2022-08-01 to 2022-11-01 in increments of 1 day).

### Use of artificial intelligence

Artificial intelligence (AI) tools including ChatGPT (GPT-5.1) and GitHub Copilot plugin for RStudio (GPT-4 and GPT-4o) were used to assist with literature review, to identify potential data sources and code development. All Large Language Model (LLM) output was checked and validated manually and modified by the authors where necessary. These tools were not used to generate data or interpret the findings, and no AI-generated content was used without human verification.

## Results

### The maximum growth rates of total and A/H3N2 influenza cases in the current season are comparable to previous seasons

We propose the epidemic growth rate as an early indicator of epidemic fitness of an influenza strain, as it is a compound measure of intrinsic transmissibility, *R*_0_, of immune escape, and of behavioural mixing patterns. The peak growth rate occurs well before the peak of cases, at which point the growth rate is zero. In this section, we compare epidemic growth rates between seasons calculated directly from reported influenza case data.

**Fig. 1:**
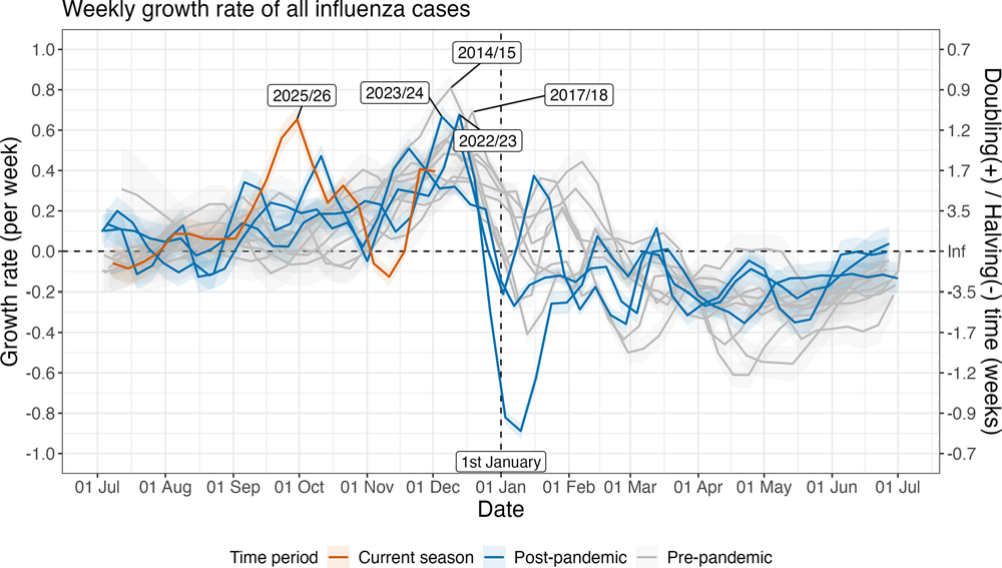
Growth rate estimates of all influenza cases from the UKHSA Respiratory DataMart. Growth rates were estimated using a Gaussian random walk model fitted to data starting in the 2011/2012 season. Coloured lines and shaded regions show posterior mean and 95% credible intervals for the model-estimated weekly growth rate. Colouring distinguishes the current season from post- and pre-pandemic seasons. Dynamics were severely disrupted during the COVID-19 pandemic and were thus excluded (2019/2020, 2020/2021 and 2021/2022 seasons). The doubling time corresponding to the growth rate is shown on the right-hand *y*-axis.

The peak growth rate of all influenza cases combined (i.e., aggregating A/H3N2, A/H1N1pdm09, unsubtyped influenza A, and influenza B) for the 2025/2026 season is comparable to previous seasons with a peak value of 0.651 (posterior mean; 95% credible intervals: 0.470-0.838) in epidemiological week 40 (2025-09-29 to 2025-10-05) (Fig. 1). The 2014/2015, 2017/2018, 2022/2023 and 2023/2024 seasons all had higher peak growth rates for influenza cases combined, though the 2023/2024 season featured a mixture of subtypes (Table 1). The 2025/2026 season stands out as an outlier in the timing of peak growth, which occurred in epidemiological week 40. In contrast, all other seasons showed peak growth rates between week 47 and week 51. After aligning the epidemic curves to the date of peak growth rate, overall trends appeared similar (Fig. S4), though the 2025/2026 season has shown a secondary, albeit smaller rise in growth rate matching the timing of other influenza seasons.

**Table 1:** Peak overall influenza and A/H3N2 growth rates starting from the 2011/2012 season

Season	Dominant subtype	Epi week of peak growth rate (all influenza)	Peak growth rate of all influenza cases	Epi week of peak growth rate (A/H3N2)	Peak growth rate of A/H3N2 cases
2014/2015	A/H3N2	50	0.812 (0.637–0.986)	50	0.840 (0.665–1.02)
2017/2018	Influenza B	51	0.694 (0.552–0.835)	51	0.618 (0.435–0.804)
2022/2023	A/H3N2	50	0.676 (0.592–0.760)	50	0.641 (0.546–0.739)
2023/2024	Mixed A/H3N2/A/H1N1pdm09	49	0.665 (0.506–0.823)	49	0.647 (0.481–0.806)
**2025/2026**	**A/H3N2**	**40**	**0.651 (0.470–0.838)**	**40**	**0.709 (0.506–0.919)**
2012/2013	Influenza B	50	0.634 (0.448–0.822)	50	0.452 (0.195–0.723)
2016/2017	A/H3N2	49	0.560(0.377–0.753)	49	0.573 (0.389–0.754)
2024/2025	A/H1N1pdm09	47	0.512 (0.372–0.648)	47	0.304 (0.114–0.492)
2011/2012	A/H3N2	49	0.482 (0.210–0.765)	49	0.55 (0.252–0.855)
2018/2019	A/H1N1pdm09	51	0.474 (0.315–0.635)	51	0.545 (0.316–0.783)
2015/2016	A/H1N1pdm09	49	0.375 (0.152–0.596)	1 (2016)	0.280 (−0.0192–0.599)
2013/2014	A/H1N1pdm09	50	0.362 (0.116–0.615)	50	0.364 (0.0832–0.673)

Note that estimates from the COVID-19 era (2019/2020, 2020/2021 and 2021/2022 seasons) have been excluded. Rows are ordered by decreasing peak overall influenza growth rate. Estimates from the 2025/2026 season are shown in bold.

Restricting our analysis to only influenza A/H3N2 cases gave similar growth rate results, though the 2025/2026 season was ranked second for peak A/H3N2 growth rate at 0.709 (0.506–0.919), surpassed only by the 2014/2015 season, which peaked at 0.840 (0.665–1.02). Similar trends were observed using the WHO FluNet data, though with a higher peak growth rate of A/H3N2 (Fig. S5).

We observed higher peak growth rates of all influenza cases in the 0–4 and 5–18 year old age groups compared to individuals aged 65+ (Fig. S6). The difference in weekly growth rates between children and adults was far higher than has been observed in the prior two influenza seasons, but comparable to the 2022/2023 season when A/H3N2 last dominated (Table 2).

**Table 2:** Differences in peak weekly growth rates of all influenza cases by age group relative to the 19–64 year-old age group

Season	0–4	5–18	65+
2022/2023	0.236	0.409	0.169
2023/2024	0.052	0.118	0.226
2024/2025	0.065	0.194	0.032
**2025/2026**	**0.203**	**0.343**	**0.116**

Values shown are the absolute difference in the mean estimated peak weekly growth rate by age group (years of age) relative to the 19–64 year-old age group. Estimates from the 2025/2026 season are shown in bold.

### Estimates for the time-varying reproduction number are comparable to previous seasons

**Fig. 2:**
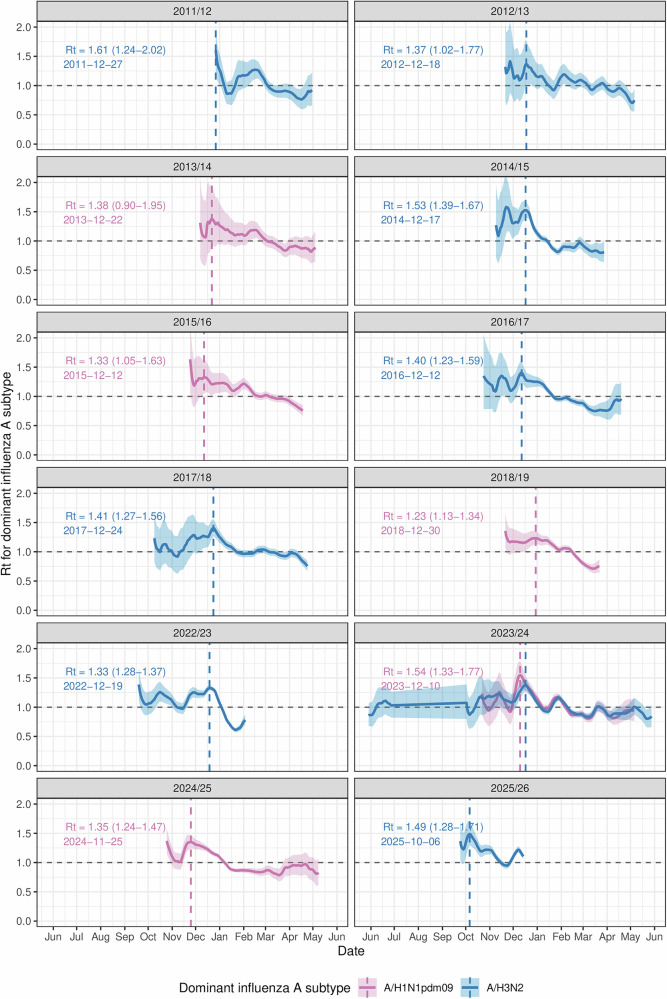
Estimates of the time-varying reproductive number for the dominant influenza A subtype in each season. Solid lines and shaded regions show the mean and 95% confidence intervals calculated using the *EpiEstim* package. The dashed vertical line shows the timing of the peak *R*_*t*_ estimate after at least 2% of total cases had been reported. The inset label shows the date and estimates of that peak *R*_*t*_ estimate. Only data for the dominant influenza A subtype in each season were used, except for the 2023/2024 season, where both influenza A/H1N1pdm09 and A/H3N2 circulated at high levels. Note that the 2012/2013 and 2017/2018 seasons were dominated by influenza B.

We used the subtype-specific case counts from the Respiratory DataMart dataset from the 2011/2012 to 2025/2026 season to estimate the time-varying reproduction number, *R*_*t*_, for the dominant influenza A subtype in each season (Figs. 2 and S7). We compared peak *R*_*t*_ values and dates occurring after at least 2% of the total number of cases for that season had occurred (to ignore early outbreak stochasticity). Peak *R*_*t*_ values occurred in December across all seasons examined, except for the 2024/2025 season (peak on 2024-11-25) and the 2025/2026 season (2025-10-06). Despite its early start, the 2025/2026 season was comparable to previous influenza seasons, with a peak *R*_*t*_ estimate of 1.49 (posterior mean; 95% credible intervals: 1.28, 1.71), compared to 1.33 (1.28–1.37) in the 2022/2023 season, 1.41 (1.27–1.56) in the 2017/2018 season, and 1.53 (1.39–1.67) in the 2014/2015 season.

### Scenario analyses using an age-stratified compartmental model

In this section, we present scenario analyses based on simulations from the age-stratified compartmental model. Note that we did not calibrate peak growth rates from the deterministic compartmental model to those estimated from the epidemiological data, and thus, we do not directly compare them. The baseline scenario generated a peak of 4540 reported symptomatic influenza cases on 2022-12-26, 19,900 symptomatic cases reported in total, and an overall final size of 16.6% of the population infected. Reported symptomatic influenza cases in the 2022/2023 season were very similar, peaking at 4510 cases on 2022-12-26, with 16,600 cumulative symptomatic cases reported overall between 2022-08-22 and 2023-02-27 (the date range of the simulation). The overall RMSE of the baseline scenario compared to reported cases was 131, which is 2.91% of the peak in reported cases (normalised RMSE). However, model fit was worse in some age groups than others: the normalised RMSE was 6.84% in the 18–65-year age group and 6.37% in the 0–5-year age group, but 10.7% in the 5–18-year age group and 19.0% in the 65+ age group. This was largely driven by the misaligned epidemic curves for these two age groups: the observed 65+ incidence curve was much later than in the model, with a reported peak on 2023-01-02. The 5–18 incidence curve was also shifted slightly later, albeit with the same peak date on 2022-12-26.

**Fig. 3:**
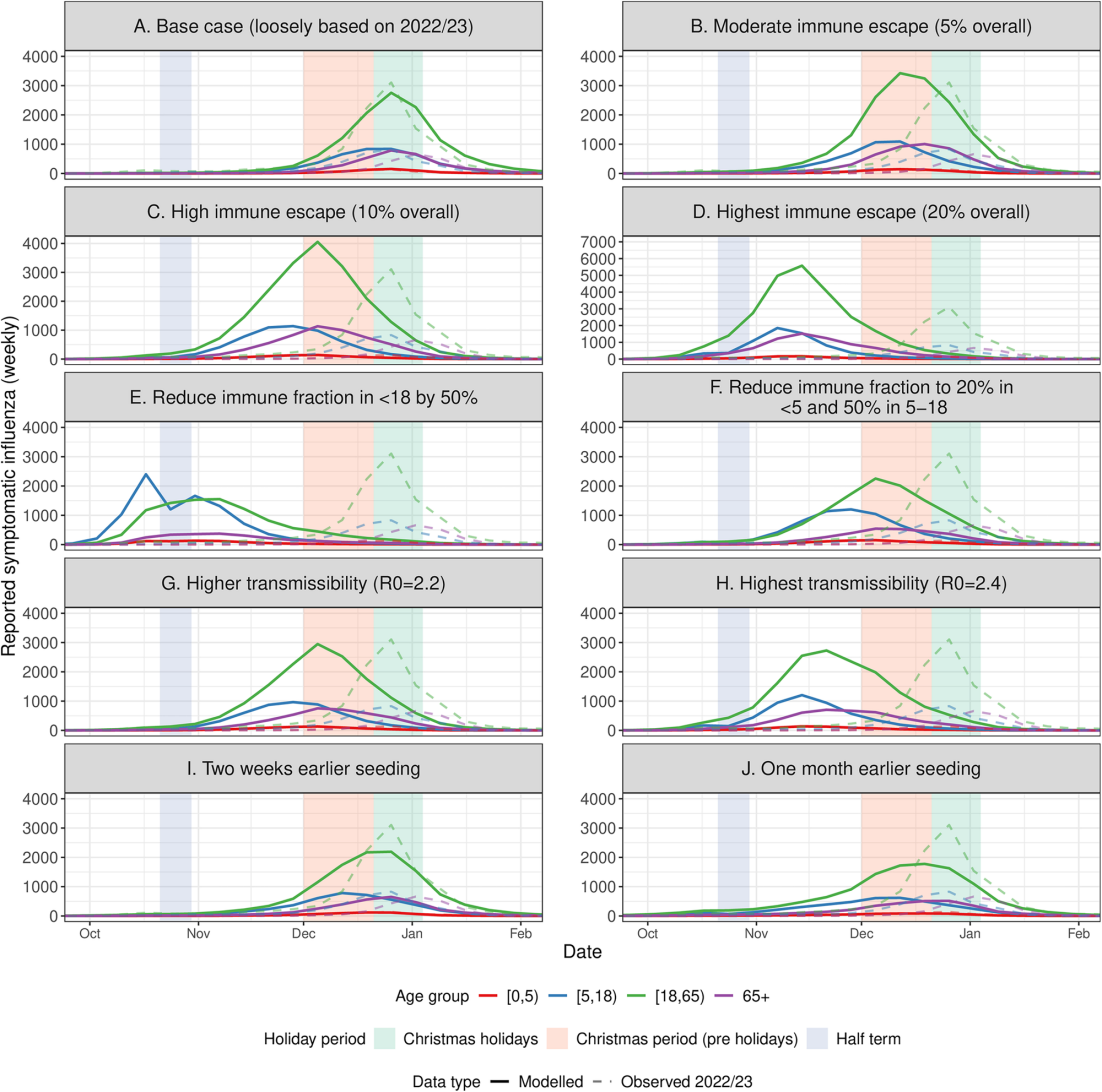
Comparison of epidemic trends across different model scenarios. Scenarios present age-stratified incidence projections under varying assumptions for A/H3N2 K clade immune escape, transmissibility and seed date. School holiday periods are highlighted in the background. Note that the base case scenario is based on visual fitting to age-stratified ILI+ data for the 2022/2023 influenza season from the RCGP RSC data (shown as dashed lines). Dynamics in the 65+ age group only are shown in Fig. S8. **A** Incidence projections from the base case scenario visually fitted to data from the 2022/2023 season. This base scenario should be interpreted with caution as no formal fitting was performed, and thus, there is considerable underrepresented uncertainty and risk of poor parameterisation. Other subplots show incidence projections assuming: **B** Moderate immune escape relative to the base scenario (*δ* = 0.95). **C** *δ* = 0.9. **D** *δ* = 0.8. **E** All initial immune fractions in <18-year-olds reduced by 50% (${R}_{0-\mathrm{4,2}}\left(0\right)=0.17$ and ${R}_{5-\mathrm{18,2}}\left(0\right)=0.30$). **F** Initial immune fraction in the 0–4 age group reduced to 20% (${R}_{0-\mathrm{4,2}}\left(0\right)=0.20$)) and the initial immune fraction in the 5–18 age group reduced to 50% (${R}_{5-\mathrm{18,2}}\left(0\right)=0.50$). **G** ${R}_{0}=2.2$. **H** ${R}_{0}=2.4$. **I** Epidemic seeding on 2025-08-24 rather than 2025-09-10. **J** Epidemic seeding on 2025-08-01 rather than 2025-09-10.

**Fig. 4:**
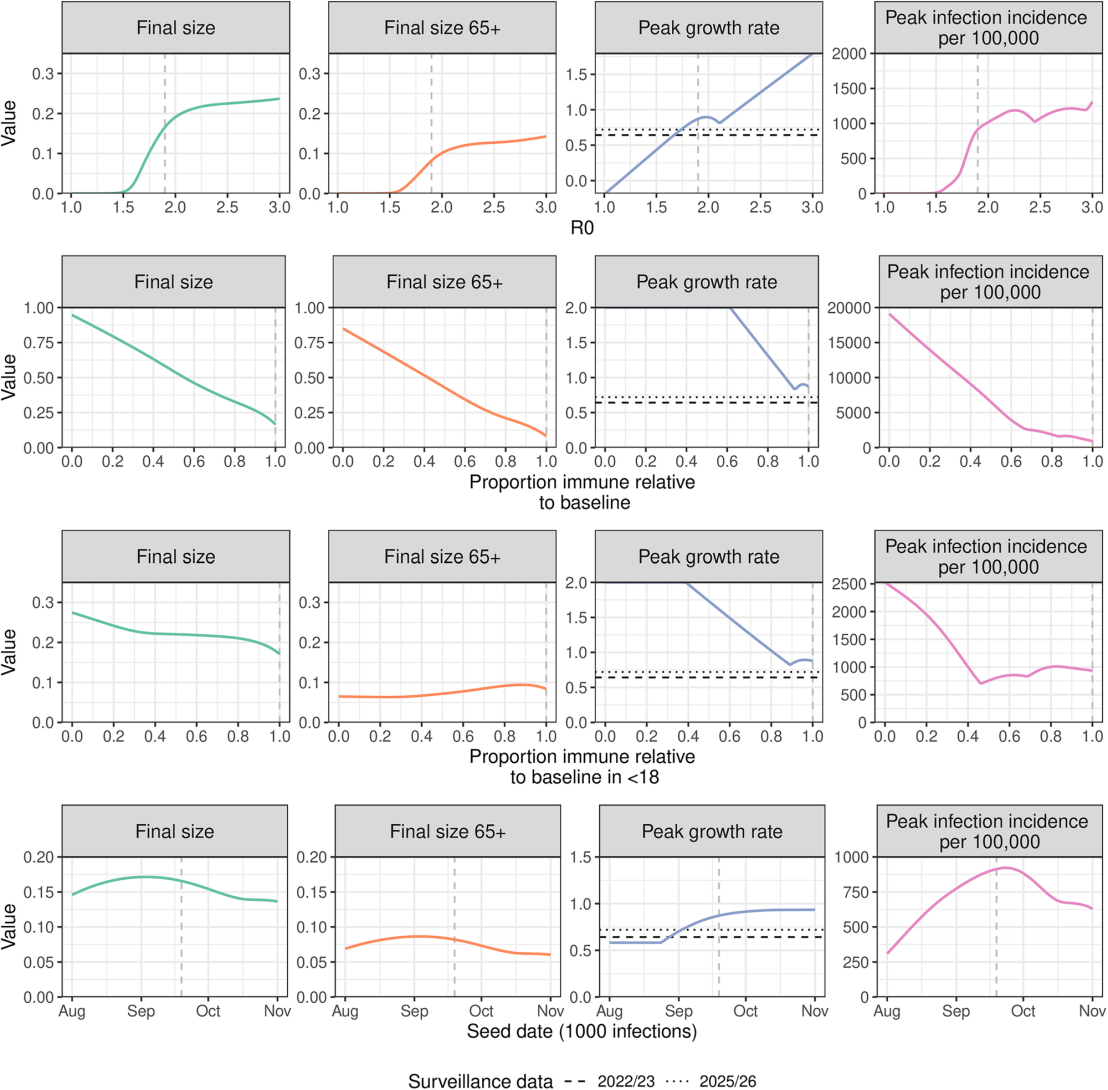
Univariable sensitivity analyses of the model outputs with respect to key parameters. Each row shows the impact of varying one parameter, given in the *x*-axis label, whilst keeping all other parameters at their baseline values. The vertical dashed line shows the values used for the base scenario. Results shown are for the epidemic final size (proportion of the population infected) overall and within the 65+ age group, and the peak weekly infection incidence. The observed peak growth rates of cases from the 2022/2023 and 2025/2026 season are shown to aid comparison. Note that the *y*-axis for “Peak growth rate” has been truncated at two to aid visualisation.

We ran 9 scenario analyses to be compared to the baseline scenario (Table 3). Scenarios B–F varied assumptions regarding immune escape. Scenarios B–D lowered overall population immunity, whereas scenarios E&F lowered population immunity only in children. Scenarios G&H assumed increased the overall transmissibility of the strain by increasing *R*_0_. Scenarios I&J assumed earlier epidemic seed times. All scenarios are shown in Fig. 3, with dynamics in the 65+ age group shown in Fig. S8, and the weekly growth rates of the scenarios shown in Fig. S9. Although we focused on individual scenarios, we also varied each of these key model parameters over a range of values individually (Fig. 4). Figure S10 shows the same scenarios compared to observed influenza case data from the 2025/2026 season as of 2026-03-30, though we emphasise that these data were not available at the time of analysis and thus were not used to calibrate the scenarios.

**Table 3:** Comparison of peak disease burden and overall disease burden of model scenarios relative to the base case

Scenario label	Scenario	Parameters changed from baseline	Peak of weekly symptomatic cases	Date of peak symptomatic cases	Relative peak growth rate	Date of peak growth rate	Cumulative symptomatic cases	Final size in 65+	Final size	Interpretation
A	Base case (loosely based on the 2022/2023 season)	–	1.00	26/12/2022	1.00	05/12/2022	1.00	1.00 (7.57%)	1.00 (16.2%)	Model parameters are roughly calibrated to match the 2022/2023 influenza season, but we note that there is considerable hidden uncertainty, strong correlations between model parameters and structural identifiability issues.
B	Moderate immune escape	Immune escape parameter, *δ*, set to 0.95	1.23	12/12/2022	0.846	07/11/2022	1.37	1.51	1.32	A relatively small loss of population immunity leads to a larger outbreak overall.
C	High immune escape	Immune escape parameter, *δ*, set to 0.90	1.39	05/12/2022	0.987	17/10/2022	1.63	1.81	1.54	As in B, but with a much larger overall impact.
D	Severe immune escape	Immune escape parameter, *δ*, set to 0.80	1.94	14/11/2022	1.56	10/10/2022	2.06	2.37	1.89	Inconsistent with observed data, as an extremely large epidemic would have already peaked in November.
E	Severe reduction in immunity in children	All initial immune fractions in <18 year olds reduced by 50% (${R}_{0-\mathrm{4,2}}\left(0\right)=0.17$ and ${R}_{5-\mathrm{18,2}}\left(0\right)=0.30$)	0.87	17/10/2022	2.05	03/10/2022	1.13	0.77	1.26	Drastically increased transmission in children would lead to a peak before the half-term holiday and the epidemic ending by the New Year. Can be ruled out.
F	Minor reduction in immunity in children	Initial immune fraction in 0–4 (${R}_{0-\mathrm{4,2}}\left(0\right)$) reduced to 20%; initial immune fraction in 5–18 (${R}_{5-\mathrm{18,2}}\left(0\right)$) reduced to 50%	0.88	05/12/2022	0.987	07/11/2022	1.13	1.00	1.20	Dampens the peak due to the half-term circuit break. Leads to a higher overall final size and a lower burden in older adults. A resurgence after half-term is expected.
G	Higher transmissibility	R_0_ increased from 1.9 to 2.2	1.04	05/12/2022	0.947	07/11/2022	1.25	1.30	1.22	An increase in final size in all age groups and an early peak, but with a similar peak size.
H	Severe transmissibility	*R*_0_ increased from 1.9 to 2.4	0.99	21/11/2022	1.35	10/10/2022	1.31	1.38	1.28	Similar to G but counterintuitively leads to a more drawn-out epidemic with slightly lower peak incidence. This is likely due to the timing of the half-term dampener with respect to incidence, which lessens the overshoot of infections after herd immunity is reached.
I	Two weeks earlier seeding	Seeding on 2025-08-24 rather than 2025-09-10	0.79	19/12/2022	0.748	05/12/2022	0.99	0.97	1.00	A very similar overall burden of infection and with a much lower peak burden expected in mid-December.
J	One month earlier seeding	Seeding on 2025-08-01 rather than 2025-09-10	0.63	19/12/2022	0.680	03/10/2022	1.00	0.99	1.00	Similar to I, but with an overall peak growth rate seen in the first week of October rather than early December.

Peak growth rate was taken as the highest overall weekly growth rate after at least 1% of all infections had occurred. All results shown are based on the ratio of outputs from the scenario to the base case. We do not report absolute numbers due to the lack of formal model fitting.

Scenarios with increased immunity escape generated earlier and larger epidemics that could peak in early December (Table 3 and Fig. 3). Reducing the initial proportion of the population in the immune compartment by just 5% (relative to the compartment’s size) leads to an increase in the overall final size of the epidemic, with cases increasing earlier in the season but still peaking in early December. Larger drops in initial population immunity lead to implausibly early and large epidemics, which is inconsistent with surveillance data available at the time of analysis, suggesting that the level of immune escape of the K clade strain may have reduced population immunity by no more than 10% if all other parameters were unchanged.

Reducing the immune fraction only in individuals under 18 years of age leads to earlier and larger epidemics, though with a reduced epidemic peak size. A major reduction in immunity in children leads to a much longer epidemic duration with peak incidence in October, but a similar total final size and peak size compared to more moderate reductions. Final size in the 65+ group is either similar or reduced. This is likely due to the interaction with the half-term period, which acts as a temporary circuit breaker to dampen the incidence curve.

Varying key model parameters individually leads to four important observations (Fig. 4). First, increased intrinsic transmissibility (proxied by increasing *R*_0_ without changing the population immune fraction) generates earlier epidemic peaks, more cases prior to the Christmas period and a larger final size. However, final size becomes saturated at higher values of *R*_0_ due to preexisting immunity in the population. Second, peak epidemic growth rates do not increase monotonically with *R*_*0*_, as the half-term school holiday can cause a dampening effect when it aligns with the early exponential phase of the epidemic. Third, at low levels of overall immune escape or immune escape in children (moving right to left in the second and third rows, third column of Fig. 4), peak growth rates do not increase monotonically, again likely due to the timing of the epidemic with respect to the half-term school holiday. Fourth, earlier epidemic seeding does not necessarily lead to a larger overall epidemic or higher peak; the seed time affects the timing of the epidemic with respect to school holidays and the Christmas period, where contact patterns and thus transmission rates change. Seeding the epidemic earlier led to a more drawn-out epidemic with a lower infection peak, but a very similar final size and timing of the peak (Fig. 3).

## Discussion

There are multiple virus properties that could explain the unusually early and rapid onset of the influenza season. In our scenario analyses, mid-season epidemiological trends were compatible with moderate levels of immune escape in all ages, or slightly greater immune escape in children, or a 10–20% higher *R*_0_, or an earlier seed date with no change in virus fitness or immune escape. Retrospective comparison of these scenarios to surveillance data up to 2026-03-30, which showed lower peak case incidence than in the 2022/2023 season, suggests that only relatively small changes to epidemiological parameters were needed to explain the earlier and more rapid season start. However, there is a wide range of parameter values and combinations thereof consistent with observed trends. In particular, *R*_0_ and immune escape are not uniquely identifiable based on epidemiological data alone, as both contribute to the effective reproduction number, *R*_eff_ and the epidemic growth rate. Thus, external immunological data are needed to disentangle the relative contribution of these two parameters, though this does not affect conclusions drawn from directly comparing empirical growth rates to past seasons.

Antigenic escape from both the vaccine strain and the overall population immune landscape is a predictor of subsequent seasonal influenza burden^4,26,27^. For the subclade K A/H3N2 viruses, substantially reduced reactivity of vaccine raised ferret antisera and human sera were observed, leading to concerns of a particularly severe season^4,30,31^. Recent serosurveys in humans support immune escape as a key factor, confirming lower (though still present) antibody recognition and neutralisation of subclade K viruses than other J.2A/H3N2 viruses in humans^32–35^. Age-stratified HAI data from Portugal suggested higher seroprevalence in children than adults^34^, providing some evidence against a scenario with greater immune escape in children than adults, though this finding is not consistent across studies^36^. HAI titre is an imperfect correlate of protection and other immune mechanisms, such as cellular immunity, are also important. Thus, the reduction in overall population immunity may be less than predicted by antibody escape alone, highlighting the need for more complete measures of population immunity for predicting the population-level impact of antigenic changes.

For seasonal influenza, which is likely driven by high within-age-group social mixing and greater susceptibility to infection in children, changes in contact patterns during school term times and holidays have a major impact on the shape of the epidemic^37,38^. In almost all modelled scenarios, an earlier and faster epidemic growth rate leads to earlier depletion of susceptibles with a dampening effect due to the half-term school holiday. Increases in transmission do not always translate to bigger epidemics because of the interaction between epidemic dynamics and school holidays that prevent epidemic ‘overshoot’. Although these changes to contact patterns are difficult to parameterise, our base model likely represents realistic shifts in behaviour^29^. Whether a new strain with enhanced transmissibility or immune escape will lead to a higher winter burden partially depends on the timing of peak transmission relative to school holidays—the half-term holiday can act as a circuit breaker, dampening the peak and spreading cases over a longer period.

The analyses performed here are high-level and use data that are routinely collected. Similar analyses were used widely during the COVID-19 pandemic, but have since become less common for seasonal respiratory diseases. We suggest that real-time growth rate characterisation and public, interactive tools for ongoing scenario analyses should be maintained regularly and updated early in the season to rule in or out different scenarios. Rapid, early epidemic growth is compatible with changes to one or more parameters relative to previous seasons, but not all combinations lead to a greater cumulative or peak infection incidence. Thus, ruling out certain scenarios is helpful for situational awareness and public health planning. Maintaining this toolkit would require low resource but consistent specialist input and real-time access to absolute reported influenza case numbers. Furthermore, combining multiple data sources with scenario analyses can narrow the range of possible epidemic curves: global surveillance and early case notification at the national level can help to understand the volume and timing of epidemic seeding^39^; routine serosurveys measuring human immune function against circulating strains can help quantify immune escape; and rapid estimation of epidemic growth rates and *R*_*t*_ from case data allow comparison of transmission rates to previous seasons.

Our analyses have several limitations. For the epidemiological analyses where we calculated growth rates and *R*_*t*_ using public ILI+ data, we used multiple data sources, each with limitations in representativeness and reporting algorithms, and our analyses required additional assumptions to align age groups and dates. When estimating growth rates, we used a Gaussian random walk model to smooth the incidence curve, which may mask true sudden changes in growth rates. Growth rate and *R*_*t*_ estimates are also likely biased by changes in testing intensity and reporting rates. We note that overconfident estimates from small sample sizes are a known issue with renewal equations, particularly affecting pre-season estimates when case numbers are very low^40^. All of the data used were reported weekly, which might obscure more finely resolved trends. Finally, as our aim was to understand epidemiological trends of subclade K viruses rather than healthcare burden, we only analysed reported influenza case data and not hospitalisations. Analyses of healthcare burden would instead need to focus on hospital admissions and occupancy data, which are more directly relevant for hospital planning.

The scenario analyses also have multiple limitations. This is a complex model with a large number of correlated parameters, making structural and practical identifiability using epidemiological data alone difficult^41,42^. We therefore did not perform formal model fitting due to time constraints, though we note that complex, age-stratified compartmental models have been successfully fitted to multiple surveillance data sources using Bayesian methods^43^. Instead, we chose plausible parameter ranges based on commonly assumed influenza parameters (*R*_0_, infectious period, final size), and then manually calibrated the parameters to achieve a reasonable fit to the 2022/2023 influenza incidence data based on four heuristics. We cannot exclude other parameter values giving comparable fits to the data, and the scenario analyses are dependent on this baseline. Assumptions regarding age-specific immunity, immune escape, and all-or-nothing immunity in two classes rather than stratified immunity all have a large impact on the projected incidence curves^44^, and we therefore recommend using the tool to inform a general understanding of the system rather than for predictions. The focus of this analysis was to understand the impact of an influenza strain with increased immune escape, transmissibility, or earlier seeding on the timing and magnitude of peak incidence, and overall proportion infected, relative to the baseline. We therefore did not attempt to directly compare the model-derived growth rates to those calculated from the epidemiological analysis.

A key omission in our model is vaccination, which we did not include due to challenges in parameterising age-specific vaccine efficacy against infection and disease. Our model is also limited by using outdated contact data and strong assumptions for behaviour changes in school holidays and the Christmas period. Although we are confident that these assumptions capture general trends, more recent and well-calibrated parameter values would improve the accuracy of the analysis. Finally, we did not consider seasonal forcing due to climate factors^45,46^. In the deterministic compartmental model, growth rates are highest at the start of the epidemic when the susceptible fraction is highest (Fig. S9). Therefore, other than the temporary changes to contact rates over school holidays and the Christmas period, the model follows a monotonic decline in transmission rates as susceptibles become depleted. In reality, factors such as temperature and relative humidity will change over time and thus may contribute to increasing growth rates early in the season.

In summary, our analysis of epidemiological data shows that the influenza A/H3N2 subclade K in England had an unusually early peak epidemic growth rate and time-varying reproduction number, but with comparable magnitude to previous severe seasons over the previous 15 years. The time-varying reproduction number peaked at 1.49 (CI = 1.28,1.71) on 2025-10-06, slightly above typical values of 1.2–1.3 reported in the literature^47^, but similar to our estimates for past seasons. Growth rates of A/H3N2 cases in children <18 years old were higher than in adults. In our scenario models, only small reductions in population immunity (the parameter *δ*) are required to drive an earlier influenza season with more cumulative infections relative to the baseline model. Larger values for immune escape led to epidemics with earlier and larger incidence peaks than were observed, suggesting that substantial escape from overall population immunity is only plausible with a compensatory reduction in intrinsic transmissibility. Overall, our findings as of mid-December 2025 suggest that the total infection incidence in England is likely at the upper end of past influenza seasons but not atypical. Epidemiological data reported since completing these analyses showed a continued decline in influenza cases and test positivity from early December 2025^43^.

## Supplementary information

Supplementary information

## Data Availability

All influenza surveillance data used in this study are publicly available. Original data sources were: (1) SGSS and Respiratory DataMart from the UKHSA public dashboard; (2) RCGP RSC from weekly reports and the ORCHID Virology Dashboard^16,18^; and (3) the WHO FluNet dashboard^13^. The raw and processed datasets used in the analyses are fully available at: https://github.com/hay-idd/influenza_H3N2_k_clade/tree/main/data. The version of the data used for the final analysis is available at: 10.5281/zenodo.20764128.

## Code availability

All analyses were conducted in R version 4.2.2. The compartmental model was implemented as an interactive Shiny app, available at https://hay-idd.shinyapps.io/ModelFluUk-H3N2/. All code required to reproduce the analysis is publicly available at https://github.com/hay-idd/influenza_H3N2_k_clade under the GNU General Public License v3.0. The version of the code used for the final analysis is available at: 10.5281/zenodo.20764128.
