# Supplementary material for: Influenza A/H3N2 epidemiology in England during the 2025 to 2026 season: a mathematical modelling study
Source: Commun Health. 2026 Aug 5;1(1):16. doi: 10.1038/s44528-026-00016-3 (PMC13441895; doi:10.1038/s44528-026-00016-3)
Supplement: Supplementary file 1 [file 44528_2026_16_MOESM1_ESM.pdf]

Supplementary Material for Influenza A/H3N2 epidemiology in England during the 2025 to 2026 season: a mathematical modelling study

Table S1. Model parameters assumed for the baseline scenario. Values shown are the default parameter values used in the baseline model scenario and default options in the interactive web tool.

Parameter	Assumed value
R_0: basic reproduction number	1.9
T_g: infectious period	4.5 days
γ: immune escape multiplier	1
Seed date	19th September
Seed size in age group 1 (0-4 yrs)	1000
Population size	60,000,000
Proportion of work contacts kept in school holidays	0.75
Multiplier for home contacts in school breaks	1.00
Multiplier for non-school and non-work contacts in school breaks	1.10
Multiplier for all non-school contacts in Christmas period (1-15 December)	1.25
Proportion of all contacts kept over Christmas	0.70
Multiplier for home contacts over Christmas holiday	3.00
Proportion initially immune (0-4 yrs)	0.35
Proportion initially immune (5-18 yrs)	0.60
Proportion initially immune (19-64 yrs)	0.70
Proportion initially immune (65+ yrs)	0.70
Symptomatic fraction and reporting rate (0-4 yrs)	0.05
Symptomatic fraction and reporting rate (5-18 yrs)	0.20
Symptomatic fraction and reporting rate (19-64 yrs)	0.30
Symptomatic fraction and reporting rate (65+ yrs)	0.50

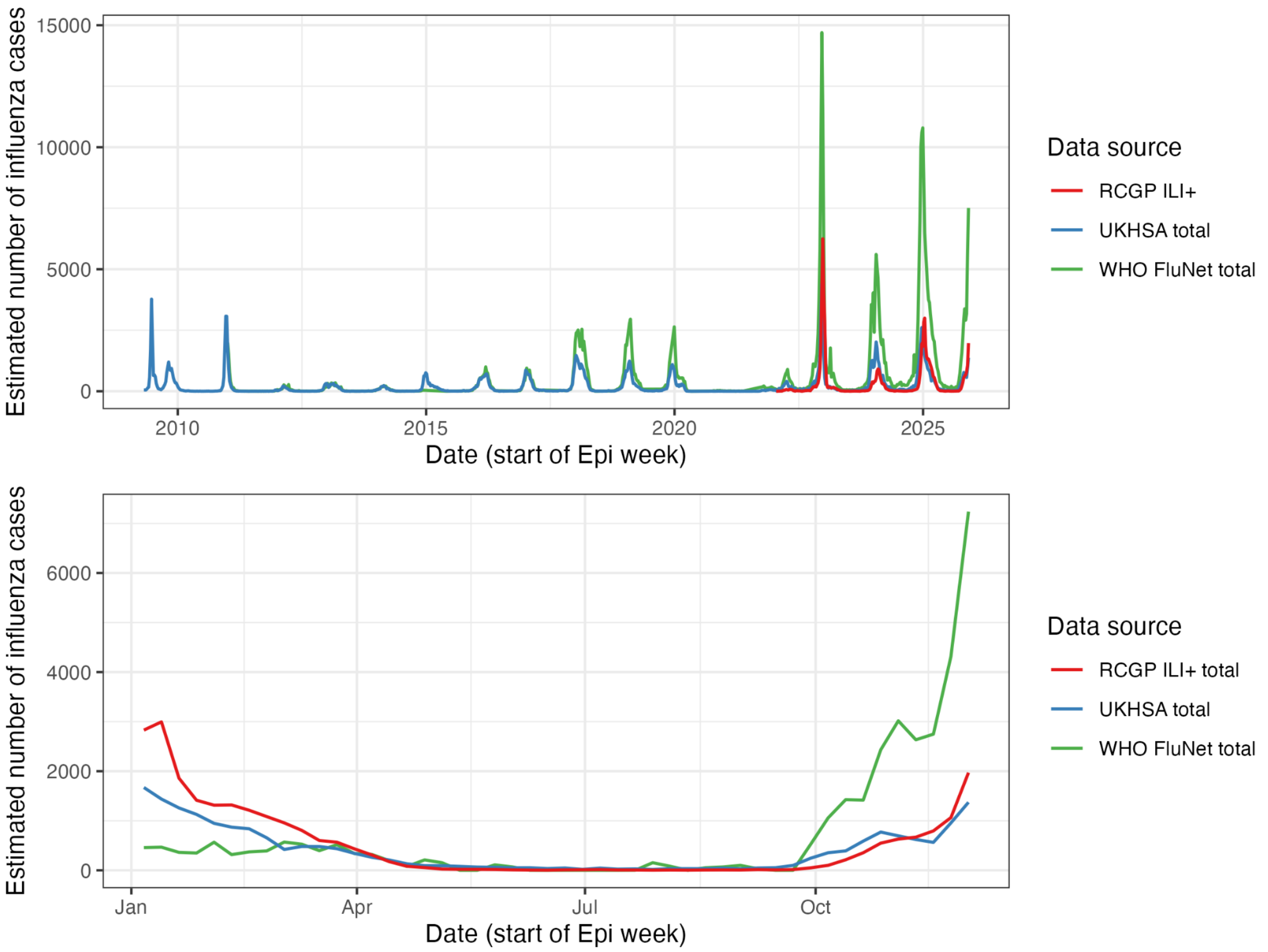

Figure S1. Comparison of the three data sources used in the analyses for all influenza cases. RCGP = Royal College of General Practitioners Research & Surveillance Centre. UKHSA = Respiratory DataMart system. The top panel shows all data. Bottom panel zooms in on the period since 2025-01-01.

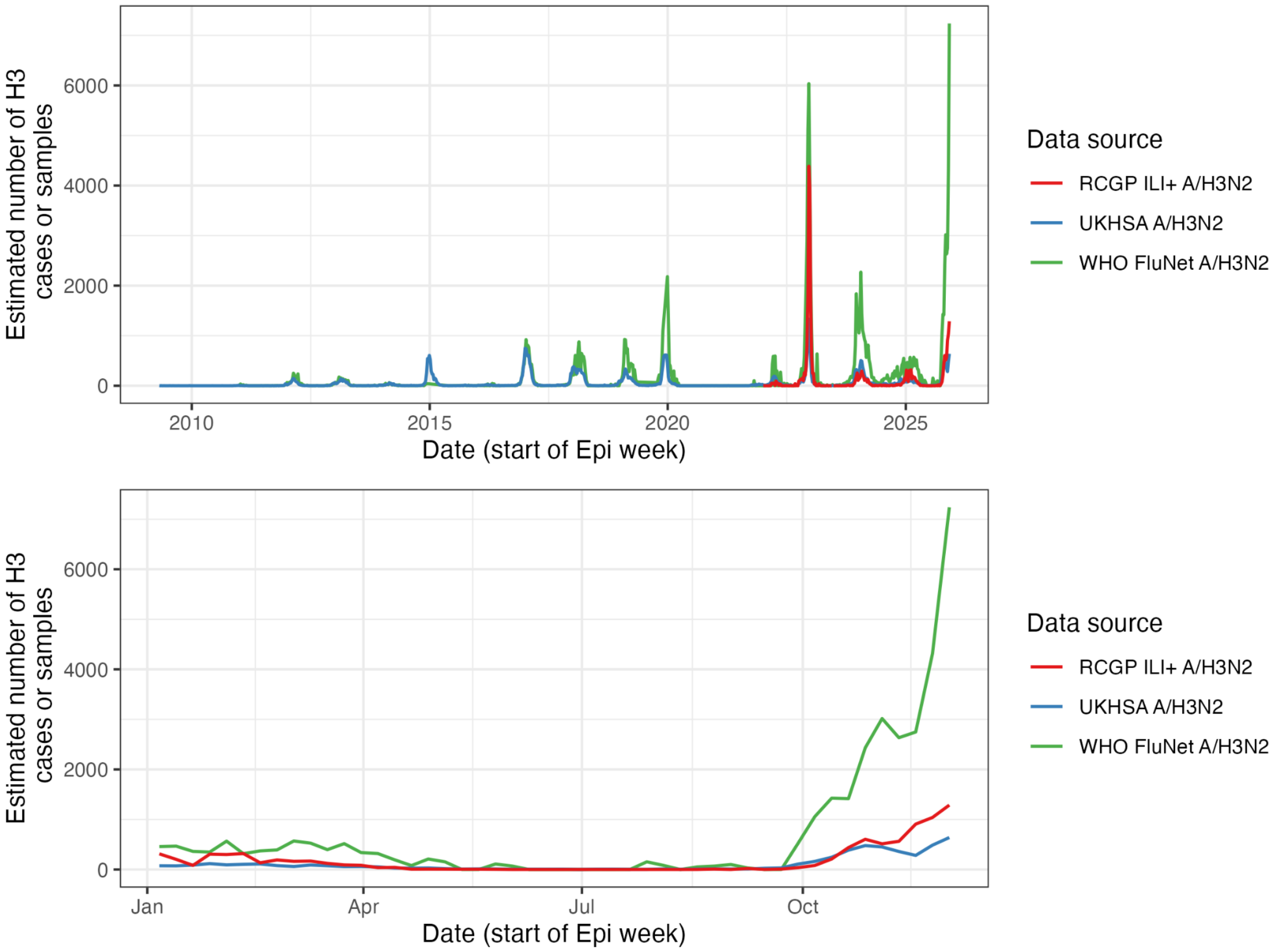

Figure S2. Comparison of the three data sources used in the analyses for influenza A/H3N2. RCGP = Royal College of General Practitioners Research & Surveillance Centre. UKHSA = Respiratory DataMart system. The top panel shows all data. Bottom panel zooms in on the period since 2025-01-01.

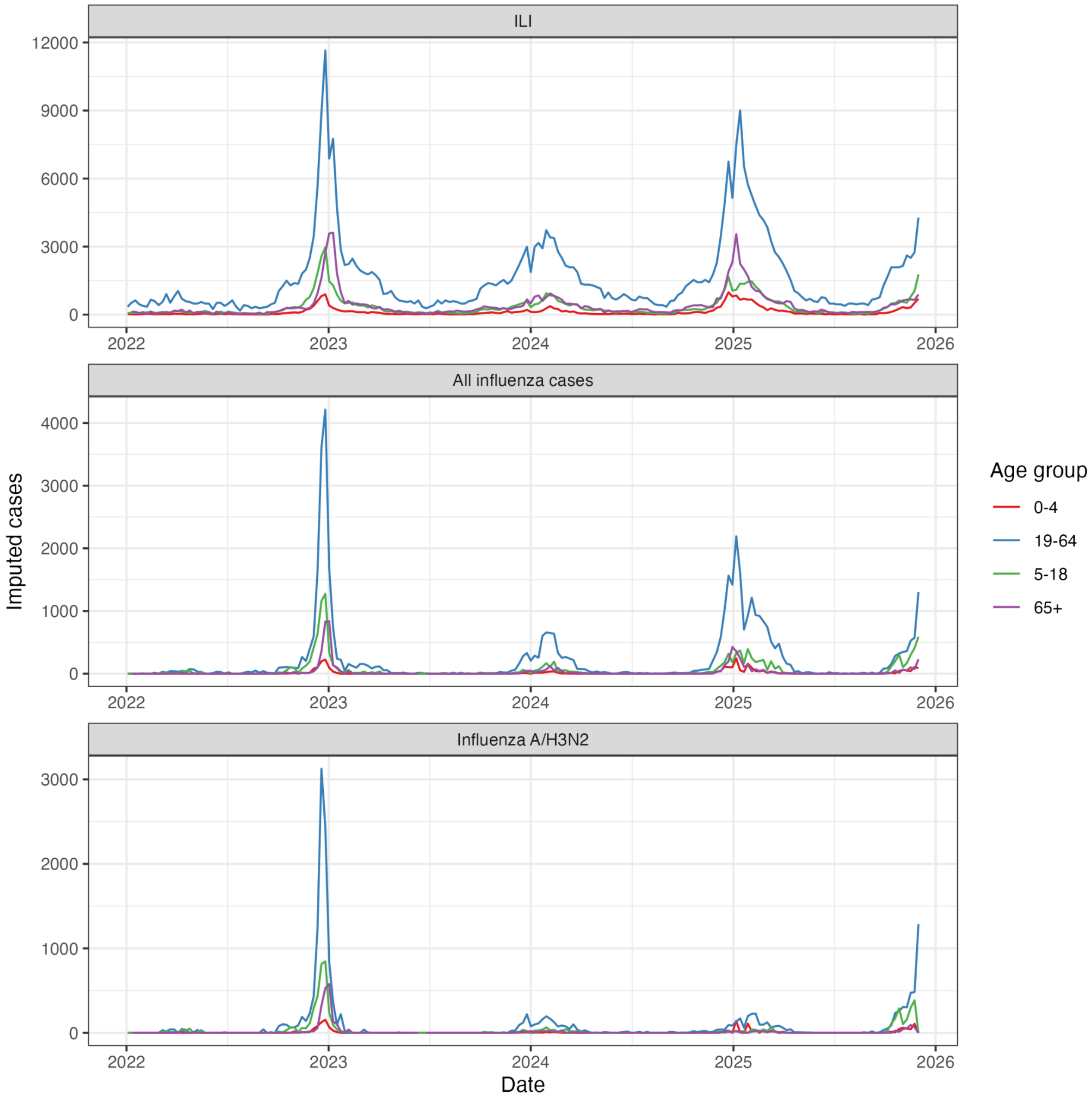

Figure S3. Comparison of age-stratified influenza surveillance indicators. Subplots show different indicators: influenza-like-illness, influenza confirmed cases, and influenza A/H3N2 confirmed cases (ILI+). Data was obtained from the RCGP RSC.

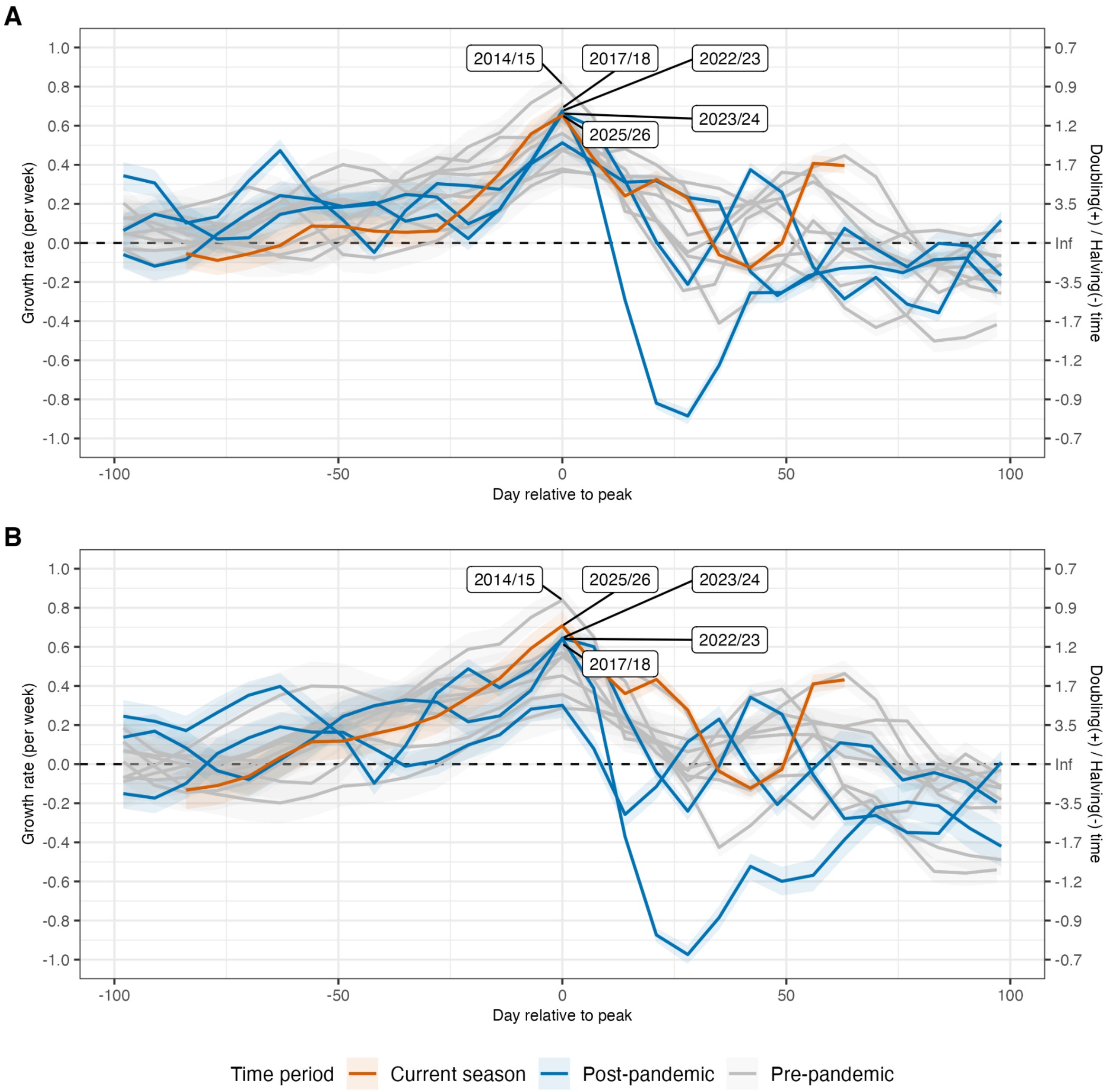

Figure S4. Growth rate estimates of all (A) and A/H3N2 (B) influenza cases from the UKHSA Respiratory DataMart using a Gaussian random walk model, starting in the 2011/12 season and aligned by date of peak growth rate. Estimates are identical to **Figure 1** but shifted to align the date of peak growth rate. Coloured lines and shaded regions show posterior mean and 95% credible intervals for the model-estimated weekly growth rate. Colouring distinguishes the current season from post and pre pandemic seasons. Dynamics were severely disrupted during the COVID-19 pandemic and were thus excluded (2019/20, 2020/21 and 2021/22 seasons). The doubling time corresponding to the growth rate is shown on the right-hand y-axis. Seasons are labelled where the peak weekly growth rate exceeded 0.6.

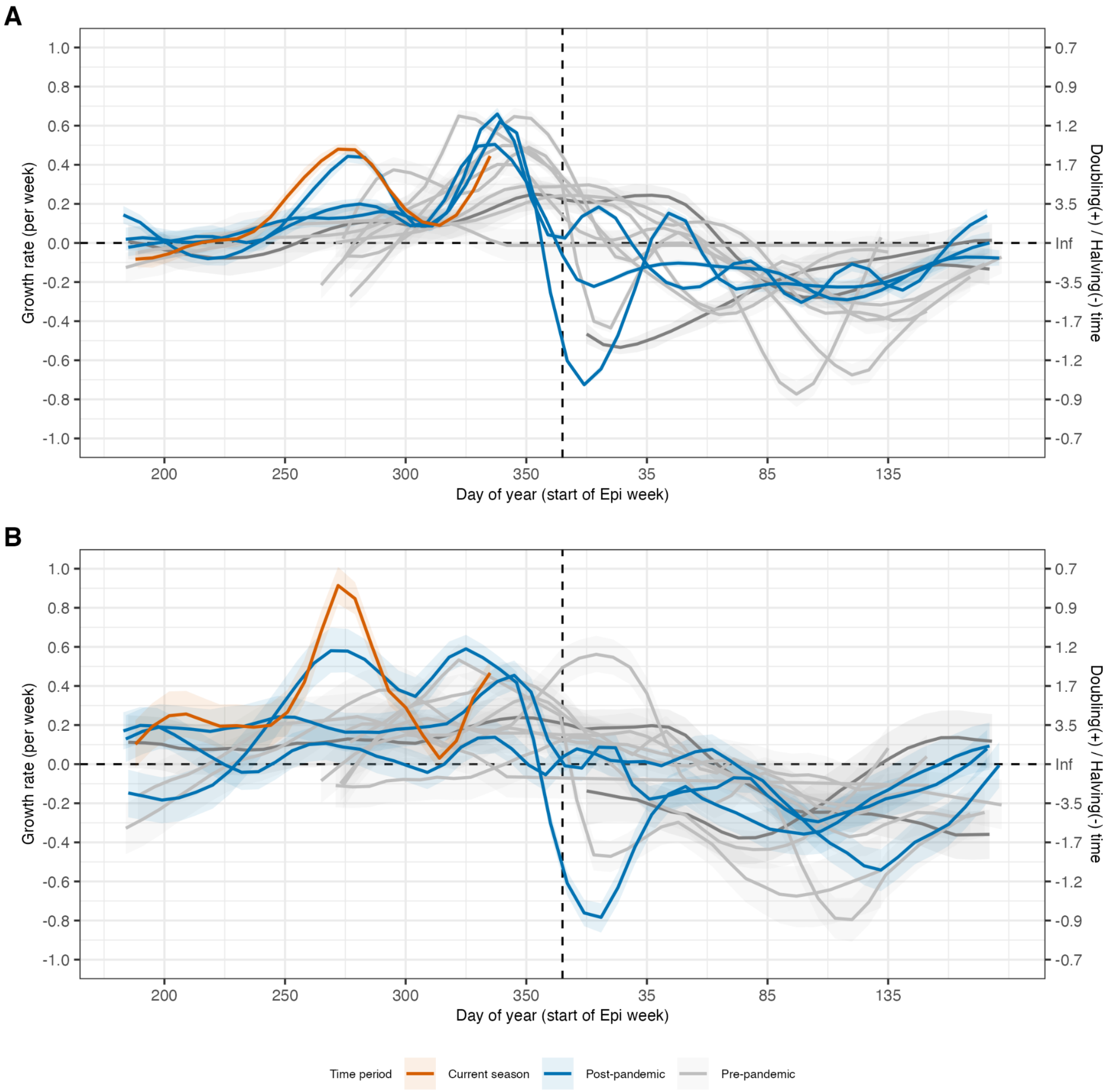

Figure S5. Growth rate of influenza cases from the WHO FluNet database. (A) All influenza cases. (B) A/H3N2 cases. Coloured lines and shaded regions show posterior mean and 95% credible intervals for the model-estimated weekly growth rate. Colouring distinguishes the current season from post, pre and during pandemic seasons. Doubling time is shown on the right-hand y-axis.

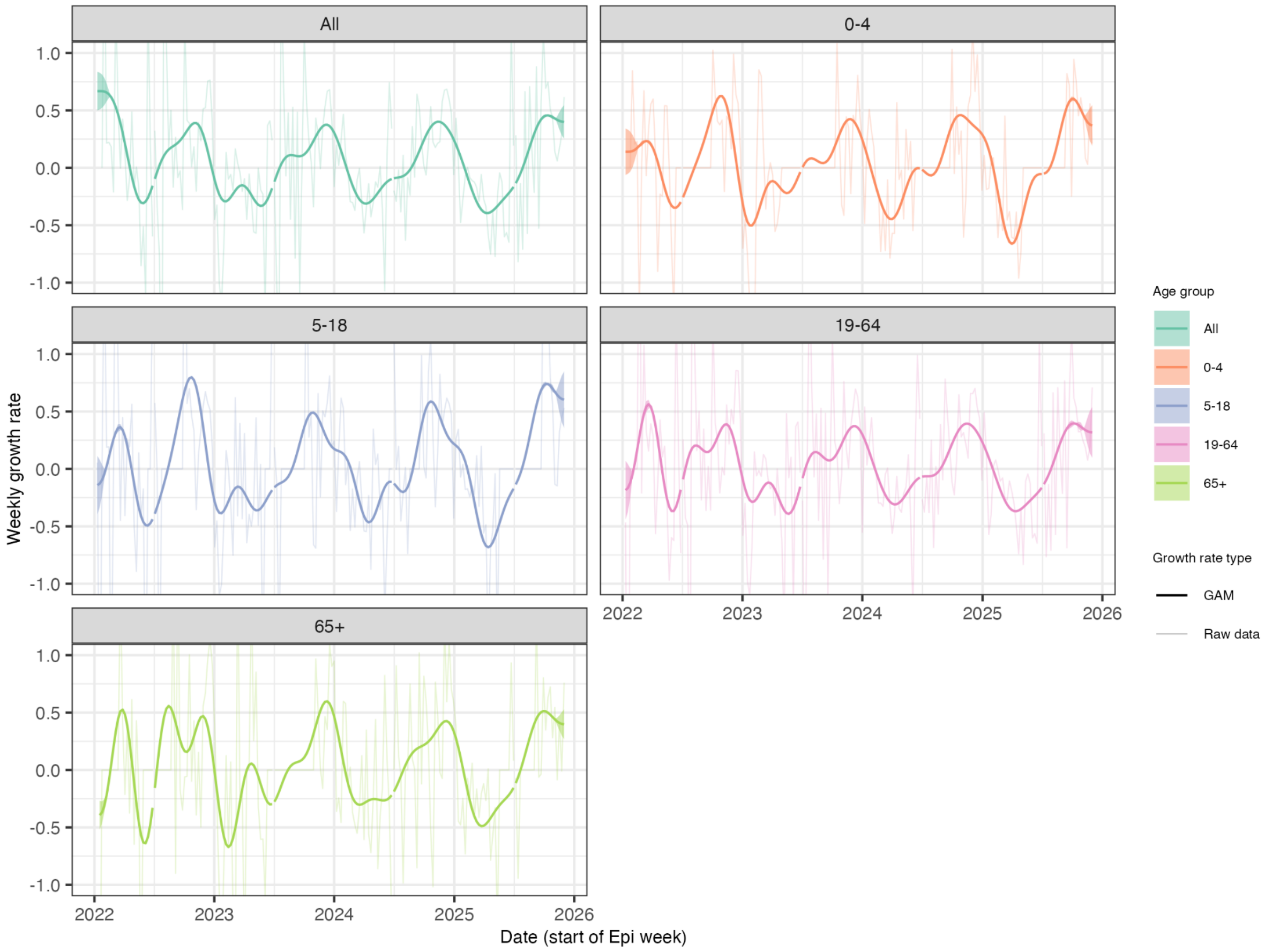

Figure S6. Growth rate estimates of age-stratified A/H3N2 cases from the ILI+ indicator. Shown are the empirical growth rates and model fits using the GAM. Each subplot shows estimates for a different age group or overall. Solid lines and ribbons show mean estimates and 95% confidence intervals.

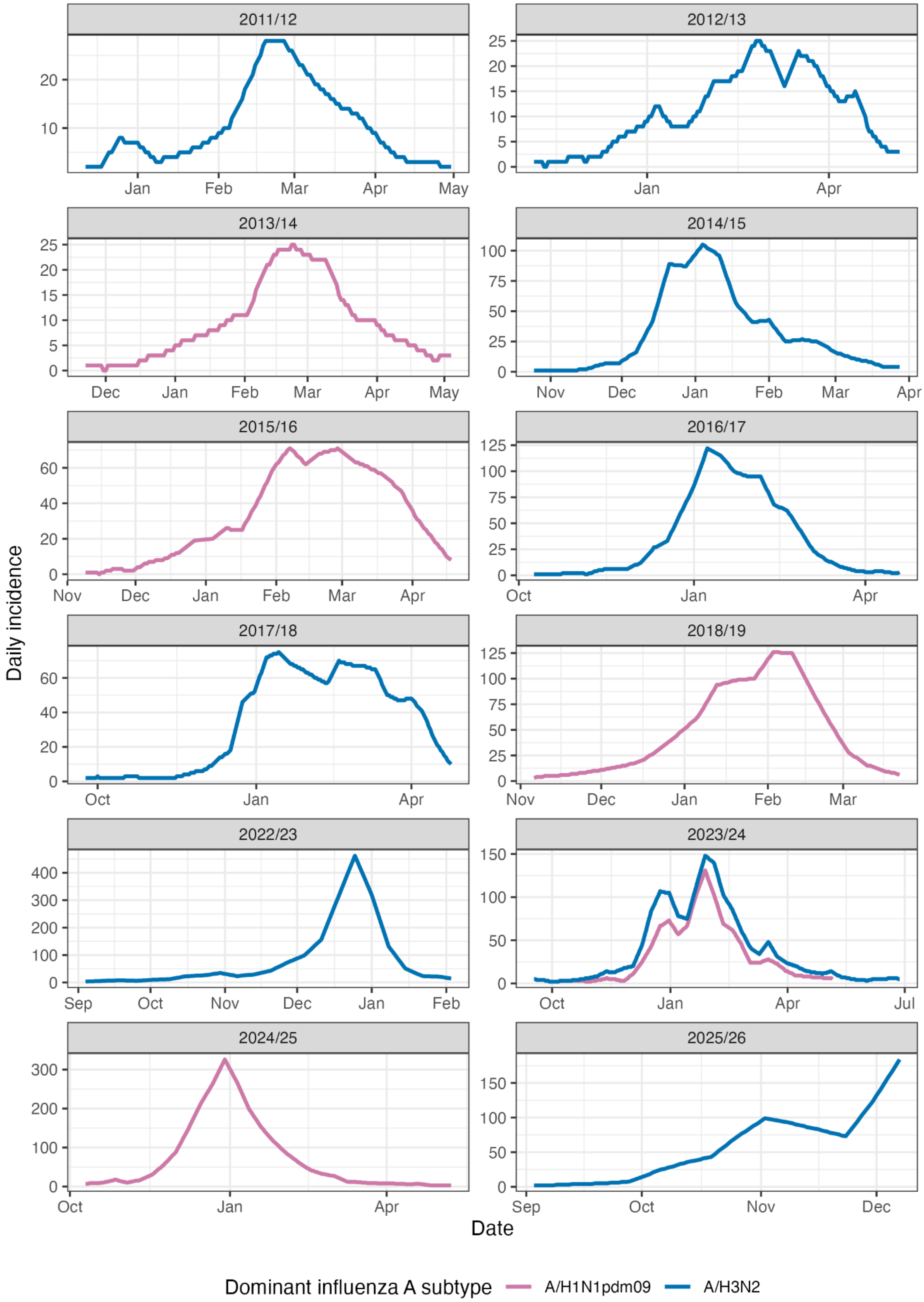

Figure S7. Daily incidence data used for R_t estimation in Figure 2. Each subplot shows approximate daily incidence of influenza cases from the UKHSA Respiratory DataMart in England from the 2011/12 to 2025/26 season. Lines are coloured based on whether the season was dominated by A/H1N1pdm09 or A/H3N2. Note that the 2023/24 season did not have a clearly dominant subtype and thus both subtypes are shown.

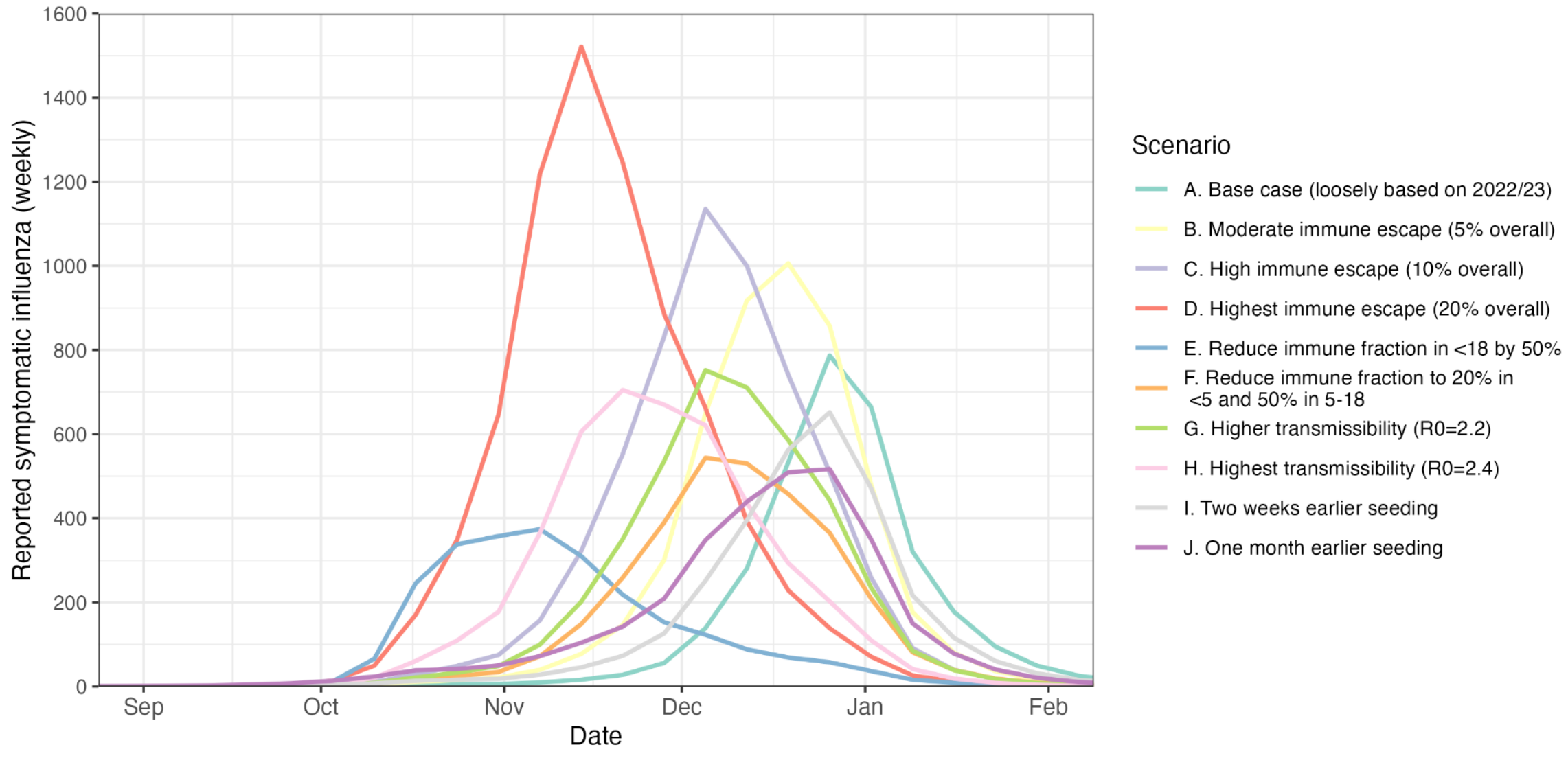

Figure S8. Comparison of symptomatic influenza incidence in the 65+ age group from the scenario analyses shown in Figure 3. Each line shows the model-projected incidence of reported symptomatic influenza infections in the 65+ age group for each scenario matching **Figure 3**.

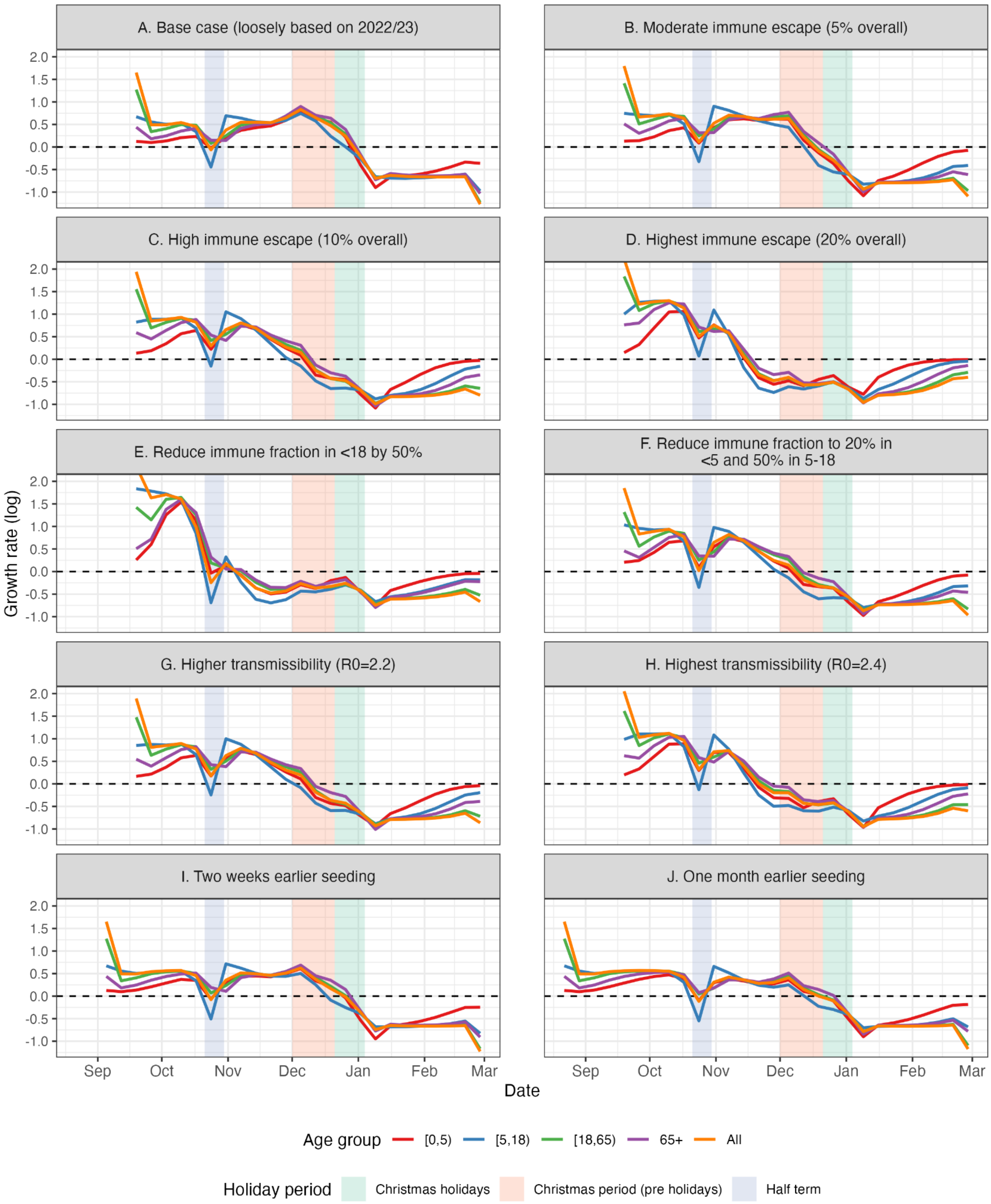

Figure S9. Comparison of weekly age-stratified growth rates from scenarios matching Figure 3. Scenarios present age-stratified weekly growth rates of infections under varying assumptions for A/H3N2 K subclade immune escape, transmissibility and seed date. School holiday periods are highlighted in the background. A growth rate of zero is shown with the dashed horizontal line. Scenarios match those in **Figure 3**, showing weekly growth rate projections for scenarios assuming**:** (A) Base case scenario manually fitted to data from the 2022/23 season. (B) Moderate immune escape relative to the base scenario (δ=0.95). (C) δ=0.9. (D) δ=0.8. (E) All initial immune fractions in <18-year-olds reduced by 50% ($R_{0-4,2}(0) = 0.17$ and $R_{5-18,2}(0) = 0.30$). (F) Initial immune fraction in the 0-4 age group reduced to 20% ($R_{0-4,2}(0) = 0.20$)) and the initial immune fraction in the 5-18 age group reduced to 50% ($R_{5-18,2}(0) = 0.50$). (G) $R_0 = 2.2$. (H) $R_0 = 2.4$. (I) Epidemic seeding on 2025-08-24 rather than 2025-09-10. (J) Epidemic seeding on 2025-08-01 rather than 2025-09-10.

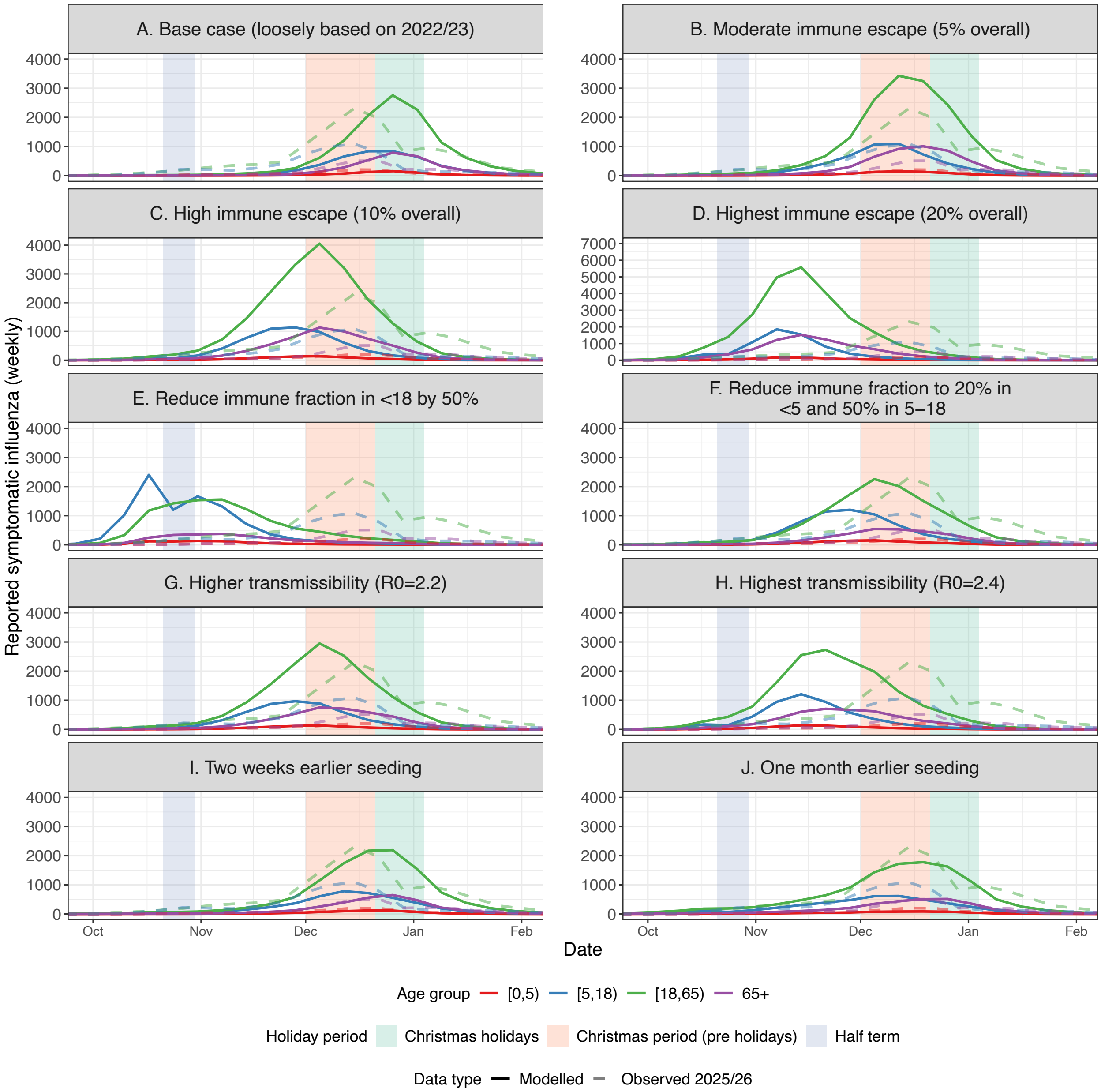

Figure S10. Model-projected epidemic trends from different scenarios compared to data from the 2025/26 influenza season. Scenarios present age-stratified incidence projections under varying assumptions for A/H3N2 K subclade immune escape, transmissibility and seed date. School holiday periods are highlighted in the background. Age-stratified ILI+ data from the 2025/26 season from the RCGP RSC data are shown as dashed lines. Scenarios match those in **Figure 3**, showing incidence projections for scenarios assuming**:** (A) Base case scenario manually fitted to data from the 2022/23 season. (B) Moderate immune escape relative to the base scenario (δ=0.95). (C) δ=0.9. (D) δ=0.8. (E) All initial immune fractions in <18-year-olds reduced by 50% ($R_{0-4,2}(0) = 0.17$ and $R_{5-18,2}(0) = 0.30$). (F) Initial immune fraction in the 0-4 age group reduced to 20% ($R_{0-4,2}(0) = 0.20$)) and the initial immune fraction in the 5-18 age group reduced to 50% ($R_{5-18,2}(0) = 0.50$). (G) $R_0 = 2.2$. (H) $R_0 = 2.4$. (I) Epidemic seeding on 2025-08-24 rather than 2025-09-10. (J) Epidemic seeding on 2025-08-01 rather than 2025-09-10.
